# Metabolite Cross-Feeding Enhances Virulence in a Model Polymicrobial Infection

**DOI:** 10.1371/journal.ppat.1002012

**Published:** 2011-03-31

**Authors:** Matthew M. Ramsey, Kendra P. Rumbaugh, Marvin Whiteley

**Affiliations:** 1 Section of Molecular Genetics and Microbiology, The University of Texas at Austin, Austin, Texas, United States of America; 2 Department of Surgery, Texas Tech University Health Sciences Center, Lubbock, Texas, United States of America; University of California, San Francisco, United States of America

## Abstract

Microbes within polymicrobial infections often display synergistic interactions resulting in enhanced pathogenesis; however, the molecular mechanisms governing these interactions are not well understood. Development of model systems that allow detailed mechanistic studies of polymicrobial synergy is a critical step towards a comprehensive understanding of these infections *in vivo*. In this study, we used a model polymicrobial infection including the opportunistic pathogen *Aggregatibacter actinomycetemcomitans* and the commensal *Streptococcus gordonii* to examine the importance of metabolite cross-feeding for establishing co-culture infections. Our results reveal that co-culture with *S. gordonii* enhances the pathogenesis of *A. actinomycetemcomitans* in a murine abscess model of infection. Interestingly, the ability of *A. actinomycetemcomitans* to utilize L-lactate as an energy source is essential for these co-culture benefits. Surprisingly, inactivation of L-lactate catabolism had no impact on mono-culture growth *in vitro* and *in vivo* suggesting that *A. actinomycetemcomitans* L-lactate catabolism is only critical for establishing co-culture infections. These results demonstrate that metabolite cross-feeding is critical for *A. actinomycetemcomitans* to persist in a polymicrobial infection with *S. gordonii* supporting the idea that the metabolic properties of commensal bacteria alter the course of pathogenesis in polymicrobial communities.

## Author Summary

Many bacterial infections are not the result of colonization and persistence of a single pathogenic microbe in an infection site but instead the result of colonization by several. Although the importance of polymicrobial interactions and pathogenesis has been noted by many prominent microbiologists including Louis Pasteur, most studies of pathogenic microbes have focused on single organism infections. One of the primary reasons for this oversight is the lack of robust model systems for studying bacterial interactions in an infection site. Here, we use a model co-culture system composed of the opportunistic oral pathogen *Aggregatibacter actinomycetemcomitans* and the common oral commensal *Streptococcus gordonii* to assess the impact of polymicrobial growth on pathogenesis. We found that the abilities of *A. actinomycetemcomitans* to persist and cause disease are enhanced during co-culture with *S. gordonii*. Remarkably, this enhanced persistence requires *A. actinomycetemcomitans* catabolism of L-lactate, the primary metabolite produced by *S. gordonii*. These data demonstrate that during co-culture growth, *S. gordonii* provides a carbon source for *A. actinomycetemcomitans* that is necessary for establishing a robust polymicrobial infection. This study also demonstrates that virulence of an opportunistic pathogen is impacted by members of the commensal flora.

## Introduction

The survival of pathogens in the human body has been rigorously studied for well over a century. The ability of bacteria to colonize, persist and thrive *in vivo* is due to an array of capabilities including the ability to attach to host tissues, produce extracellular virulence factors, and evade the immune system. Invading pathogens must also obtain carbon and energy from an infection site, and specific carbon sources are required for several pathogens to colonize and persist in the host [1]. Although mono-culture infections provide interesting insight into pathogenesis, many bacterial infections are not simply the result of colonization with a single species, but are instead a result of colonization with several [2], [3], [4], [5]. The mammalian oral cavity is an excellent environment to study polymicrobial interactions as it is persistently colonized with diverse commensal bacteria as well as opportunistic pathogens. Our lab has utilized a two-species model system composed of the opportunistic pathogen *Aggregatibacter actinomycetemcomitans* and the common commensal *Streptococcus gordonii* to provide mechanistic insight into how specific carbon sources impact disease pathogenesis in polymicrobial infections [6], [7].

*A. actinomycetemcomitans* is a Gram-negative facultative anaerobic bacterium that inhabits the human oral cavity and is a proposed causative agent of localized aggressive periodontitis [8]. *A. actinomycetemcomitans* is found between the gums and tooth surface in the subgingival crevice [9], [10], an area restricted for O_2_ depending on tissue depth [11] and irrigated by a serum exudate called gingival crevicular fluid (GCF). GCF not only contains serum proteins such as complement and immunoglobulin [12], but also glucose from 10 to 500 µM in healthy patients [13] and as high as 3 mM in patients with periodontal infections [14]. L-lactate is produced by host lactate dehydrogenase in GCF [15], [16] and resident oral streptococci. Together glucose and L-lactate represent two of the small number of carbon sources that *A. actinomycetemcomitans* is able to catabolize [17]. *A. actinomycetemcomitans* has been proposed to primarily inhabit the aerobic [9] “moderate” pockets (4 to 6 mm in depth) of the gingival crevice as opposed to deeper anaerobic subgingival pockets [18].

In addition to *A. actinomycetemcomitans*, the subgingival crevice is home to a diverse bacterial population, including numerous oral streptococci [19], that reside in surface-associated biofilm communities [20]. Oral streptococci, aside from *Streptococcus mutans*, are typically non-pathogenic and depending upon the human subject and method of sampling, comprise approximately 5% [21] to over 60% [22] of the recoverable oral flora. Through fermentation of carbohydrates to L-lactate and sometimes H_2_O_2_, acetate, and CO_2_, oral streptococci such as *S. gordonii* have been shown to influence the composition of oral biofilms [19], [20], [23], [24]. Additionally, *S. gordonii*-produced H_2_O_2_ influences interactions between *A. actinomycetemcomitans* and the host by inducing production of ApiA, a factor H binding protein that inhibits complement-mediated lysis [7], [25]. Thus, streptococcal metabolites are important cues that influence the growth and population dynamics of oral biofilms and how oral bacteria interact with the host.

*A. actinomycetemcomitans* preferentially catabolizes L-lactate over high energy carbon sources such as glucose and fructose in multiple strains, despite the fact that this bacterium grows more slowly with L-lactate [6]. Given this preference for a presumably inferior carbon source and the observation that *A. actinomycetemcomitans* resides in close association with oral streptococci [26], [27], we hypothesize an *in vivo* benefit exists for *A. actinomycetemcomitans* L-lactate preference. To test this hypothesis, we investigated the importance of *A. actinomycetemcomitans* L-lactate catabolism during mono-culture and co-culture with *S. gordonii in vitro* and in a murine abscess model of infection. Our results reveal that co-culture with *S. gordonii* enhances colonization and pathogenesis of *A. actinomycetemcomitans*, and the ability to utilize L-lactate as an energy source is essential for these co-culture benefits. Surprisingly, inactivation of L-lactate catabolism had no impact on mono-culture growth *in vitro* and *in vivo* suggesting that *A. actinomycetemcomitans* L-lactate catabolism is only critical for establishing co-culture infections. Taken together, these results provide compelling mechanistic evidence that the metabolic properties of human commensals such as *S. gordonii* can alter the course of pathogenesis in polymicrobial communities.

## Results

### *A. actinomycetemcomitans* metabolism of glucose and L-lactate

**Figure 1:**
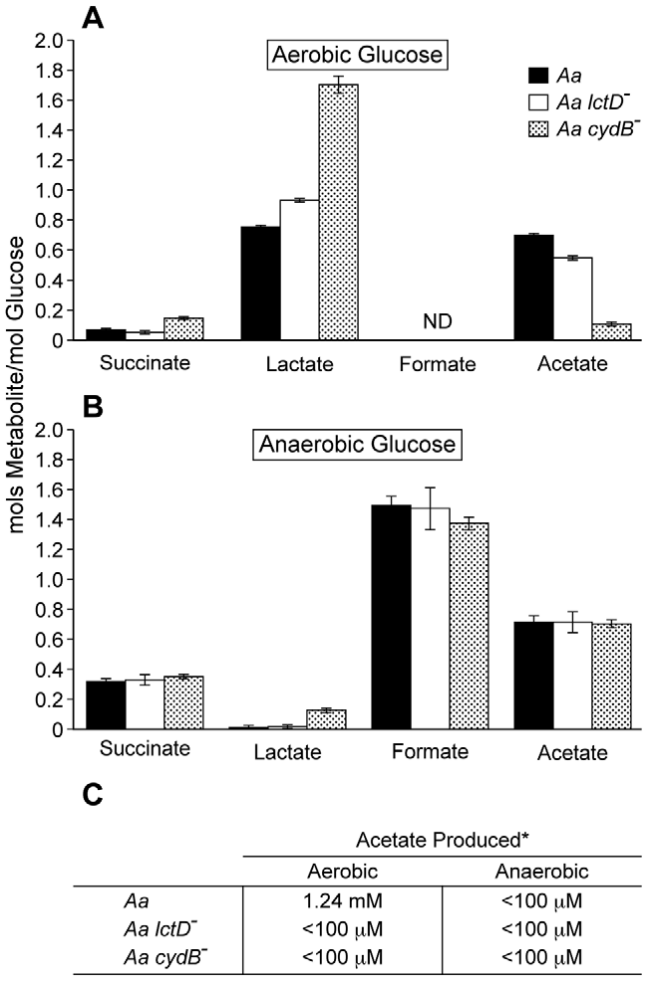
Aerobic and anaerobic metabolites produced by *A. actinomycetemcomitans*, *A. actinomycetemcomitans lctD* ^-^ and *A. actinomycetemcomitans cydB* ^-^. Resting cell suspensions of each culture were incubated (**A** )aerobically in glucose; (**B**), anaerobically in glucose; (**C**), aerobically or anaerobically in lactate. Metabolite concentrations were measured by HPLC. Data in A and B is presented as moles of metabolite produced/mole of glucose consumed. Only trace concentrations (<50 µM) of ethanol were observed in anaerobic suspensions. Error bars represent 1 standard error of the mean, n = 3. * Acetate concentrations are shown per mM of L-lactate consumed. The detection limit for acetate was 100 µM.

Within the gingival crevice, host-produced glucose and L-lactate are present [13], [14], [15], [16], [28] and likely serve as *in vivo* carbon sources for *A. actinomycetemcomitans*. However in contrast to glucose, L-lactate is also produced by the oral microbial flora, primarily oral streptococci [20]. Indeed, the ability of *A. actinomycetemcomitans* to catabolize streptococcal-produced L-lactate has been demonstrated previously [6], and it was proposed that *A. actinomycetemcomitans* consumes streptococcal-produced L-lactate during co-culture. To assess the importance of *A. actinomycetemcomitans* L-lactate catabolism in polymicrobial communities *in vitro*, we examined the metabolic profile during catabolism of L-lactate and glucose under aerobic and anaerobic conditions. Aerobically, *A. actinomycetemcomitans* primarily produced lactate and acetate from glucose (Fig. 1A) while acetate was the sole metabolite produced by L-lactate-grown bacteria (Fig. 1C). It was intriguing that lactate was produced, but not consumed, by *A. actinomycetemcomitans* during aerobic catabolism of glucose. We hypothesized that the lactate produced by *A. actinomycetemcomitans* was likely D-lactate, which is not catabolized by *A. actinomycetemcomitans* [29]. Using an enzymatic assay [30], we were able to verify that >99% of the lactate produced by *A. actinomycetemcomitans* was indeed D-lactate.

Anaerobically from glucose, *A. actinomycetemcomitans* primarily produced the mixed acid fermentation products formate and acetate along with lactate, succinate, and trace amounts of ethanol (Fig. 1B). Surprisingly, *A. actinomycetemcomitans* was unable to catabolize L-lactate anaerobically (Fig. 1C), even if the potential alternative electron acceptors nitrate or dimethyl sulfoxide were added, suggesting that L-lactate oxidation was O_2_ dependent. This is distinct from other oral bacteria including members of the genus *Veillonella* [24], [31], in which L-lactate is an important anaerobic carbon and energy source. If O_2_ respiration was indeed required for *A. actinomycetemcomitans* growth with L-lactate, we hypothesized that elimination of the terminal respiratory oxidase, which is required for aerobic respiration, would abolish L-lactate utilization by *A. actinomycetemcomitans* aerobically. To test this hypothesis, *cydB*, which encodes a component of the sole putative *A. actinomycetemcomitans* respiratory oxidase, was insertionally inactivated. The *cydB* mutant was unable to catabolize L-lactate aerobically supporting the hypothesis that L-lactate oxidation requires O_2_ respiration (Fig. 1C). Interestingly when grown with glucose aerobically, the *cydB* mutant doubled much slower (6.6 hr) than the wt (1.9 hr) and cell suspensions produced a metabolite profile that differed from the wt (Fig. 1A) indicating that while not required for aerobic growth on glucose, O_2_ respiration is the primary means by which glucose is catabolized by wt *A. actinomycetemcomitans*. As expected, the *cydB* mutant exhibited identical growth rates anaerobically on glucose (not shown) and produced similar metabolites as the wt (Fig. 1B). Collectively, these data indicate that O_2_ respiration is required for L-lactate oxidation in *A. actinomycetemcomitans*.

As the ultimate goal of this study is to assess the importance of *A. actinomycetemcomitans* L-lactate catabolism for establishing co-culture with oral streptococci, it was important to assess whether eliminating the ability of *A. actinomycetemcomitans* to utilize L-lactate affected growth with glucose. To examine this, we examined growth and metabolite production in an *A. actinomycetemcomitans* strain in which the catabolic L-lactate dehydrogenase LctD, which is present in all strains sequenced to date [32], [33], was insertionally inactivated [29]. LctD oxidizes L-lactate to pyruvate and is required for *A. actinomycetemcomitans* growth with L-lactate as the sole energy source [29]. As expected, the *lctD* mutant was unable to catabolize L-lactate aerobically or anaerobically (Fig. 1C); however, metabolite production from glucose was not affected (Fig. 1A&B) nor was the growth rate with glucose (not shown). These data indicate that L-lactate catabolism can be eliminated in *A. actinomycetemcomitans* without affecting growth and metabolite production with glucose.

### Utilization of L-lactate enhances co-culture growth

**Figure 2:**
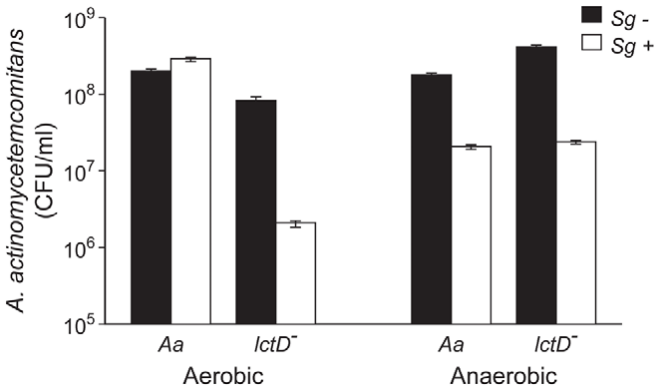
Growth of *A. actinomycetemcomitans*, *A. actinomycetemcomitans lctD* ^-^, and *S. gordonii* in aerobic and anaerobic co-cultures. Strains were grown as mono- or co-cultures in 3 mM glucose aerobically or anaerobically for 10 or 12 h respectively, serially diluted and plated on selective media to determine colony forming units per ml (CFU/ml). *A. actinomycetemcomitans* mono-culture strains are black bars and co-culture with *S. gordonii* are white bars. Error bars represent 1 standard error of the mean, n = 3.

Because *A. actinomycetemcomitans* preferentially catabolizes L-lactate in lieu of hexose sugars [6], we hypothesized that L-lactate cross-feeding was important for establishing co-culture with oral streptococci grown on glucose. To test this hypothesis, we examined growth of glucose-grown *A. actinomycetemcomitans* and *S. gordonii* during *in vitro* co-culture aerobically and anaerobically. Aerobically, wt *A. actinomycetemcomitans* co-culture cell numbers were similar to those observed in mono-culture while the *A. actinomycetemcomitans lctD* mutant exhibited an approximate 25-fold decrease in cell number during co-culture with *S. gordonii* (Fig. 2). Anaerobically, both wt *A. actinomycetemcomitans* and *A. actinomycetemcomitans lctD* ^-^ cell numbers diminished nearly 10-fold in co-culture compared to mono-culture (Fig. 2), likely due to the inability to catabolize *S. gordonii*-produced L-lactate.

**Figure 3:**
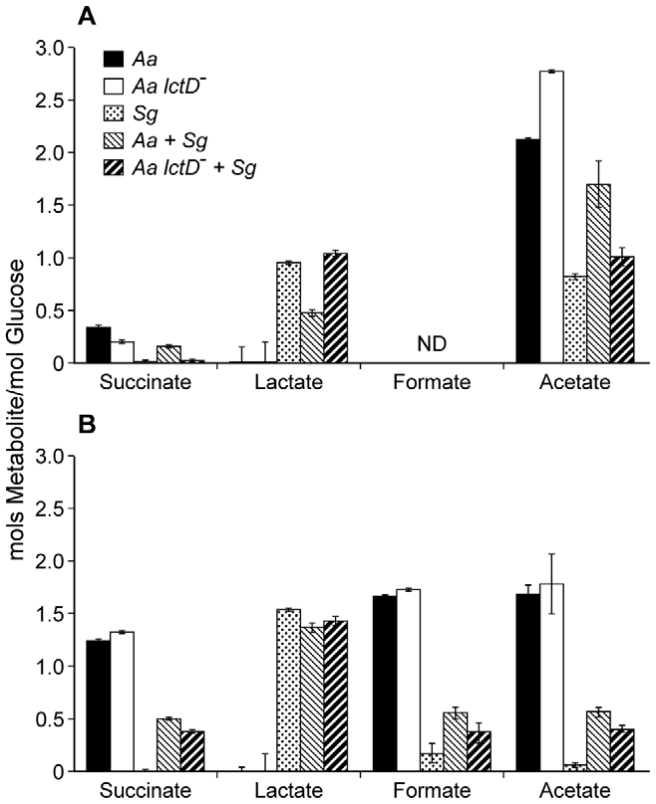
Metabolite production by *A. actinomycetemcomitans*, *A. actinomycetemcomitans lctD* ^-^, and *S. gordonii* in aerobic or anaerobic co-cultures. Supernatants of the cultures used for CFU measurements in Fig. 2 were analyzed by HPLC for metabolite production from (**A**), aerobic or (**B**), anaerobic cultures. Data is presented as moles of metabolite produced/mole of glucose consumed. Error bars represent 1 standard error of the mean, n = 3. ND  =  No Data.

Examination of aerobic metabolic end products of the *A. actinomycetemcomitans lctD^-^*/*S. gordonii* co-culture revealed high levels of lactate, reminiscent of *S. gordonii* mono-cultures, indicating that as expected, the *A. actinomycetemcomitans lctD* mutant is unable to catabolize L-lactate in co-culture (Fig. 3A). Additionally, metabolite concentrations in anaerobic co-cultures were similar to *S. gordonii* mono-culture (Fig. 3B). It should be noted that these metabolites were measured from growing cells, not cell suspensions as in Fig. 1. These data provide strong evidence that the inability to use L-lactate, even when glucose is present, significantly inhibits *A. actinomycetemcomitans* growth and survival in co-culture.

Interestingly, an approximate 7-fold increase in *S. gordonii* cell numbers were observed in the presence of *A. actinomycetemcomitans* aerobically, indicating that *A. actinomycetemcomitans* enhances *S. gordonii* proliferation under these co-culture conditions even when *A. actinomycetemcomitans* is unable to utilize L-lactate (Fig. S1 in Text S1). Importantly, the pH of the medium used in these experiments remained at neutrality; thus changes in cell numbers were not due to alterations in pH.

### L-lactate consumption is required for co-culture growth of *A. actinomycetemcomitans in vivo*

**Figure 4:**
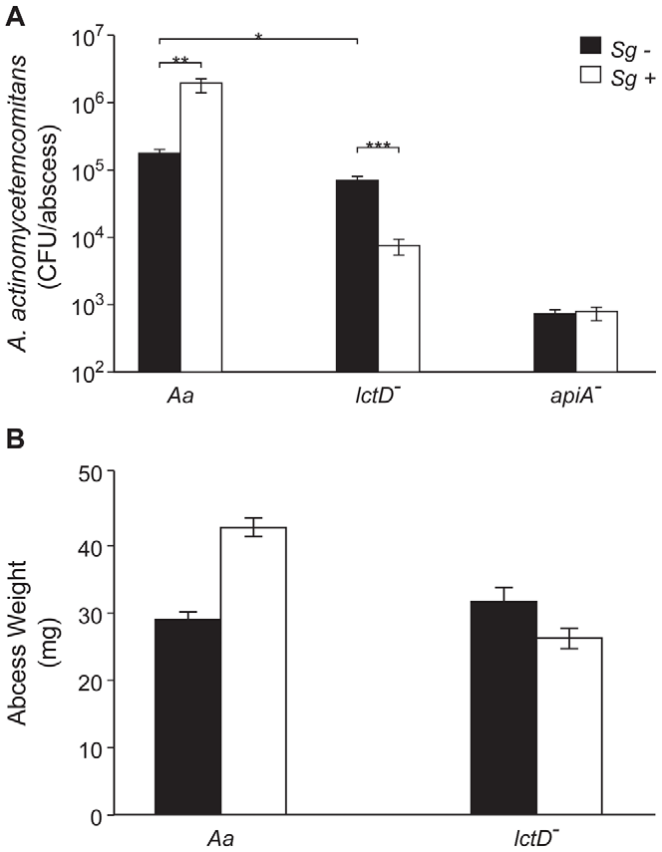
Persistence of *A. actinomycetemcomitans*, *A. actinomycetemcomitans lctD* ^-^, and *A. actinomycetemcomitans apiA* ^-^ in mono- or co-culture in a murine abscess model. **A.** Bacterial colony forming units per abscess. Wilcoxon signed-rank test values are: * p<0.02, ** p<0.01, *** p<0.008. **B.** Abscess weights 6 days post-inoculation. Error bars represent 1 standard error of the mean, n = 9. p<0.05 for wt *A. actinomycetemcomitans* in mono- and co-culture via Student's t-test.

The observation that L-lactate catabolism is critical for *A. actinomycetemcomitans* to establish co-culture with *S. gordonii in vitro* provides new insight into this model polymicrobial community; however, whether the requirement for this catabolic pathway extended to *in vivo* co-culture was not known. To examine the role of *A. actinomycetemcomitans* L-lactate catabolism for *in vivo* growth in mono- and co-culture, we used a mouse thigh abscess model. This model has relevance as *A. actinomycetemcomitans* causes abscess infections outside of the oral cavity in close association with other bacteria [34] and has been used as a model system to examine pathogenesis of several oral bacteria [35], [36]. Using this model, bacterial survival and abscess formation was assessed for wt *A. actinomycetemcomitans* and *A. actinomycetemcomitans lctD* ^-^ during mono- and co-culture with *S. gordonii* (Fig. 4).

Unexpectedly, wt *A. actinomycetemcomitans* and the *lctD* mutant established similar infections in terms of cell number (Fig. 4A) and in abscess weight (Fig. 4B) indicating that host-derived L-lactate is not an important *in vivo* nutrient source during mono-culture infection. Interestingly, wt *A. actinomycetemcomitans* displayed a 10-fold increase in cell number when co-cultured with *S. gordonii*, while cell number of the *lctD* mutant declined >100-fold compared to the wild-type providing evidence that the ability to catabolize L-lactate is crucial for *A. actinomycetemcomitans* co-culture survival *in vivo.* These data also indicate that while not critical for mono-culture growth, L-lactate is an important energy source during co-culture infection. Unlike the *in vitro* experiments (Fig. S1 in Text S1), *S. gordonii* numbers were not statistically different in monoculture or in co-culture abscesses (2.7×10^7^ and 1.3×10^7^ CFU/ml respectively; p = 0.15 via Mann-Whitney test) indicating that *S. gordonii* does not receive a benefit, at least in regard to cell number, from co-culture with *A. actinomycetemcomitans*. As a control, *in vivo* growth of the *A. actinomycetemcomitans apiA* mutant, which is hypersusceptible to killing by innate immunity, was examined. As expected, the *apiA* mutant exhibited a >250-fold decrease in mono-culture *in vivo* survival, which was unchanged in the presence of *S. gordonii* (Fig. 4A).

## Discussion

Microbes within polymicrobial infections often display synergistic interactions that result in enhanced colonization and persistence in the infection site [5], [34], [36], [37], [38], [39], [40]. Such interactions have been particularly noted in oral polymicrobial infections, although the molecular processes controlling these synergistic interactions are not well defined. Detailed mechanistic studies of the interactions required for enhanced persistence *in vivo* is a critical step towards a more comprehensive understanding of natural polymicrobial infections. In this study, we used a model polymicrobial infection [6], [7] to determine the importance of metabolic cross-feeding for establishing co-culture infections. Cross-feeding in polymicrobial populations has been reported in numerous studies [24], [41], [42], but its importance for establishing co-culture infections has not been investigated in depth. The methodology used in this study began with detailed studies of the metabolic pathways required for growth with the *in vivo* carbon sources glucose and L-lactate, followed by examination of the importance of specific catabolic pathways for establishing co-culture infections.

It is relevant to discuss the rationale for two *in vivo* experimental parameters: using a ‘smooth’ strain of *A. actinomycetemcomitans* in lieu of a ‘rough’ strain; and using a murine abscess model in lieu of a rat periodontal infection model [43], [44]. A “smooth” strain of *A. actinomycetemcomitans*, which displays impaired surface attachment, was used in this study [45], [46]. As we were not investigating attachment or biofilm development, we opted to utilize a smooth strain that had undergone robust metabolic characterization, and feel this decision is justified as this bacterium clearly causes abscess infections in this model (Fig. 4). The murine abscess model was used for several reasons. First, in addition to periodontal infections, *A. actinomycetemcomitans* causes abscess infections outside of the oral cavity that resemble, from a gross morphological standpoint, the abscess model infection [34]; thus the abscess model has clinical relevance. Second, the abscess model avoids complications arising from the normal flora, which are not completely eradicated in the periodontal rat infection models, and whose presence would make interpretation of metabolic interactions extraordinarily complex. Third, the abscess model allows direct, controlled inoculation with a finite number of cells that can be quantified throughout the infection by assessing colony forming units after removal of the entire abscess [37], [47], [48]. Finally, although the abscess model has primarily been used to study anaerobic pathogens [35], [36], it is also relevant for studying aerobic pathogens, demonstrated by the large abscesses [48] formed by the strict aerobe *Acinetobacter baumanii* [17], [49]. The presence of aerobic microenvironments in the abscess is also supported by our observations that the *S. gordonii spxB* mutant is significantly impaired for abscess formation (Fig. S2 in Text S1). The *spxB* gene encodes pyruvate oxidase which utilizes O_2_ for biosynthesis of the virulence factor H_2_O_2_ [50]; thus its importance is limited to aerobic infections.

The observation that *A. actinomycetemcomitans* requires O_2_ to catabolize L-lactate was surprising, as many oral bacteria grow on L-lactate anaerobically [24], [31]. These results also solve an apparent contradiction in the literature. It was reported by multiple sources [17], [51] that *A. actinomycetemcomitans* does not catabolize L-lactate, yet we recently provided evidence that several strains of *A. actinomycetemcomitans* grow aerobically with L-lactate as the sole energy source [6], [29]. Interrogation of the previous growth environments revealed that *A. actinomycetemcomitans* was grown under very low or O_2_ free conditions; thus it is not surprising that significant growth was not observed in these studies. The O_2_ dependency of L-lactate oxidation also highlights another facet of our *in vivo* data. In the murine abscess model, the *A. actinomycetemcomitans* wt and *lctD* mutant grew equally well in mono-culture (Fig 4). However, in co-culture only the survival of the *lctD* mutant was impaired. This result is reminiscent of our *in vitro* data (Fig. 2) suggesting that O_2_ dependent metabolism occurs in our model polymicrobial infection.

**Figure 5:**
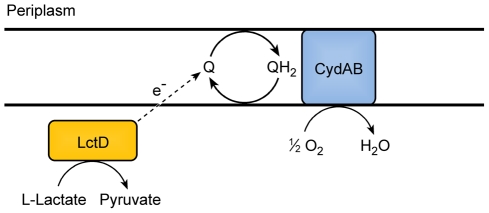
Model for electron transport during L-lactate oxidation in *A. actinomycetemcomitans*. *A. actinomycetemcomitans* requires O_2_ for oxidation of L-lactate. LctD may donate electrons from L-lactate directly to the quinone pool or utilize an unknown intermediate electron carrier represented by the dotted arrow. The cytochrome oxidase CydAD ultimately donates the electrons to O_2_.

The observation that the terminal oxidase CydB is required for aerobic growth with L-lactate allows development of a new model for L-lactate consumption in *A. actinomycetemcomitans* (Fig. 5). Since L-lactate dehydrogenase (LctD) is necessary for lactate oxidation and does not use NAD^+^ as an electron acceptor [29], anaerobic fermentation pathways that regenerate NAD^+^ cannot act as electron acceptors for L-lactate oxidation. The model predicts that *A. actinomycetemcomitans* instead donates electrons directly to the quinone pool which in turn is re-oxidized by CydAB [52]. It should be noted that this does not rule out an unknown electron carrier between LctD and the membrane associated quinone.

**Figure 6:**
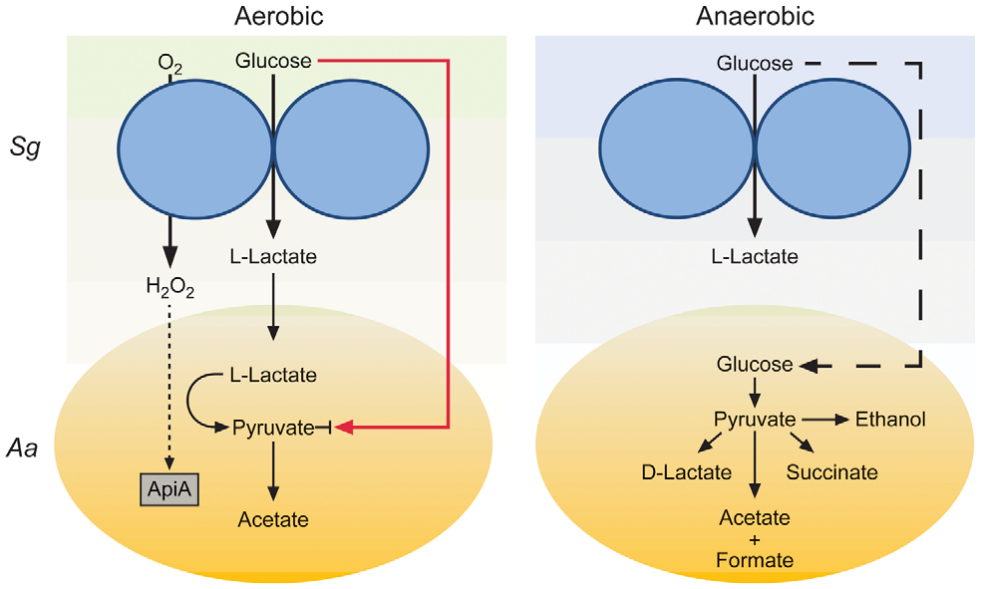
Model for enhanced persistence of *A. actinomycetemcomitans* during aerobic co-culture with *S. gordonii*. During co-culture aerobic growth with glucose, *S. gordonii* produces L-lactate and H_2_O_2_ which inhibit *A. actinomycetemcomitans* glucose uptake (red line) and induce *apiA* expression (dotted line) respectively. The production of L-lactate provides *A. actinomycetemcomitans* with a preferred carbon source for growth and reduces the need to compete with *S. gordonii* for glucose during aerobic co-culture. During anaerobic co-culture, *S. gordonii* also produces L-lactate but *A. actinomycetemcomitans* is unable to catabolize this carbon source due to the absence of O_2_; thus requiring *A. actinomycetemcomitans* to compete directly with *S. gordonii* for glucose (dashed line).

The most exciting observation from these studies is that L-lactate catabolism is likely an important factor for *A. actinomycetemcomitans* to establish a polymicrobial, but not mono-culture, infection in a murine abscess model (Fig. 4). These data indicate that host-produced L-lactate is not a vital energy source for *A. actinomycetemcomitans* in mono-culture abscesses, but when *S. gordonii* is present, L-lactate catabolism becomes critical. We speculate that in the absence of *S. gordonii,* carbohydrates such as glucose are present in the infection site for *A. actinomycetemcomitans* growth. When *S. gordonii* is introduced, competition for these carbohydrates increases, and *A. actinomycetemcomitans* is likely at a disadvantage due to its relatively slow growth and catabolic rates compared to *S. gordonii* [6]. Thus, the ability to preferentially utilize L-lactate, the primary metabolite produced by *S. gordonii,* allows *A. actinomycetemcomitans* to avoid competition with *S. gordonii* for carbohydrates and consequently enhance its survival in the abscess. This model (Fig. 6) suggests that the importance of individual carbon catabolic pathways is dependent on the context of the infection, specifically if oral streptococci are present.

Our work demonstrates that metabolic pathways required for *A. actinomycetemcomitans* proliferation during mono-culture infection are distinct from those required for co-culture infection with a common commensal. This study provides strong evidence that simply because elimination of a catabolic pathway does not elicit a virulence defect in mono-species infection does not preclude it from being important in polymicrobial infections. Since metabolic interactions can potentially occur in virtually any polymicrobial infection, our results suggest that in some cases, the ability to cause infection will be as dependent on metabolic interactions as it is on known immune defense mechanisms and classical virulence factors. Our observations also have therapeutic implications, as development of small molecule inhibitors of metabolic pathways, particularly pathways restricted to prokaryotic pathogens, have promise as new therapeutic targets. Based on this study, efforts to develop such therapeutics will require a detailed understanding of how polymicrobial cross-feeding affects colonization and persistence in an infection site.

## Materials and Methods

### Ethics statement

This study was carried out in strict accordance with the recommendations in the Guide for the Care and Use of Laboratory Animals of the National Institutes of Health. The protocol was approved by the Institutional Animal Care and Use Committee of Texas Tech University Health Sciences Center (Protocol Number: 09039).

### Strains and media

*A. actinomycetemcomitans* strains VT1169 [53], *Streptococcus gordonii* strain Challis DL1.1 (ATCC 49818), *S. gordonii spxB* ^-^ [50], *Escherichia coli* DH5α-λpir, and *E. coli* SM10-λpir were used in this study. *A. actinomycetemcomitans* and *S. gordonii* were routinely cultured using Tryptic Soy Broth + 0.5% Yeast Extract (TSBYE). For resting cell suspension *A. actinomycetemcomitans* metabolite analysis, a Chemically Defined Medium (CDM) [6] lacking nucleotides, amino acids, pimelate and thioctic acid (to eliminate further cell growth) containing either 20 mM glucose or 40 mM L-lactate was used. For co-culture experiments, complete CDM with 3 mM glucose was used. Aerobic culture conditions were 37°C in a 5% CO_2_ atmosphere shaking at 165 RPM, and anaerobic culture conditions were static growth at 37°C in an anaerobic chamber (Coy, USA) with a 5% H_2_, 10% CO_2_ and 85% N_2_ atmosphere. *E. coli* strains were grown on Luria-Bertani (LB) medium at 37°C. Where applicable, antibiotics were used at the following concentrations: chloramphenicol, 2 µg/ml for *A. actinomycetemcomitans* and 20 µg/ml for *E. coli*; spectinomycin, 50 µg/ml for selection and 10 µg/ml for maintenance for *A. actinomycetemcomitans* and *E. coli* and 100 µg/ml for selection and maintenance for *S. gordonii spxB* ^-^; kanamycin, 40 µg/ml for selection and 10 µg/ml for maintenance; naladixic acid, 25 µg/ml; streptomycin, 50 µg/ml for selection and 20 µg/ml for maintenance. For quantifying CFU/ml in co-culture assays, vancomycin (5 µg/ml) was added to agar plates to enumerate *A. actinomycetemcomitans* and streptomycin (100 µg/ml) was added to agar plates to enumerate *S. gordonii*.

### DNA and plasmid manipulations

DNA and plasmid isolations were performed using standard methods [54]. Restriction endonucleases and DNA modification enzymes were purchased from New England Biolabs. Chromosomal DNA from *A. actinomycetemcomitans* was isolated using DNeasy tissue kits (Qiagen), and plasmid isolations were performed using QIAprep spin miniprep kits (Qiagen). DNA fragments were purified using QIAquick mini-elute PCR purification kits (Qiagen), and PCR was performed using the Expand Long Template PCR system (Roche). DNA sequencing was performed by automated sequencing technology using the University of Texas Institute for Cell and Molecular Biology sequencing core facility.

### *A. actinomycetemcomitans apiA* mutant construction

Allelic replacement of *apiA* (AA2485) was carried out by double homologous recombination. For construction of the knockout construct, 856 bp and 842 bp DNA fragments flanking *apiA* were amplified and combined with the *aphA* gene (encoding kanamycin resistance) from pBBR1-MCS2 [55] by overlap extension PCR [56]. The construct was prepared so that *aphA* was positioned between the upstream and downstream regions. Primers used were: Kan-5′ (ATGTCAGCTACTGGGCTATCTG) and Kan-3′ (ATTTCGAACCCCAGAGTCCCGC) for the 1074 bp *aphA*-containing fragment; ApiA-UF (CCGATAACAGTAAGATCTTCTAC) and ApiA-UR (CAGATAGCCCAGTAGCTGACAT CCTTTTCGGCTTGAATTTATACC) for the upstream *apiA* fragment; and ApiA-DF (GCGGGACTCTGGGGTTCGAAAT GCGGTCAGAATTTTAGGTGTTTT) and ApiA-DR (CGAAACCAACGAACTCTTTATTC) for the downstream *apiA* fragment. Underlined sequences indicate overlapping DNA sequences between the *apiA* fragments and *aphA*. The overlap extension product was TA-cloned into the pGEM-T Easy vector (Promega, USA) and excised by EcoRI digest. The EcoRI fragment containing the overlap extension product was ligated into the unique EcoRI site within the λpir-dependent suicide vector pVT1461 [57]. The cloned construct, pVT1461-apiA-KO, was first transformed into *E. coli* DH5α-λpir then into *E. coli* SM10-λpir for conjugation into *A. actinomycetemcomitans*. Conjugation was performed as described [53] and potential mutants were plated onto TSBYE agar plates containing kanamycin to select for recombinant *A. actinomycetemcomitans* and nalidixic acid to kill the *E. coli* donors. Kanamycin resistant, spectinomycin sensitive double recombinants were selected and verified by PCR. Enhanced susceptibility of the *apiA* mutant to serum was verified as described previously [7].

### *A. actinomycetemcomitans cydB* mutant construction

Insertional mutagenesis of the *cydB* gene was performed by single homologous recombination using a 543 bp internal piece of the *cydB* (AA2840) gene amplified using the primers cydB-KO5′ (GAAGATCTTTATGATTAATACTATCGCGCCG) and cydB-KO3′ (GAAGATCTCAAAACCATCTTTGAAAGATAACCA). Underlined sequences represent BglII restriction sites. The internal *cydB* fragment was digested with BglII and ligated into the *A. actinomycetemcomitans* suicide vector pMRKO-1 (see below) to generate pMRKO-cydB. pMRKO-cydB was transformed into *E. coli* SM10-λpir and conjugated into *A. actinomycetemcomitans*. *A. actinomycetemcomitans* recombinants were grown anaerobically on TSBYE agar containing spectinomycin and naladixic acid. Colonies were subcultured anaerobically on liquid medium at the same antibiotic concentrations and insertion into *cydB* was verified by PCR.

### pMRKO-1 suicide vector construction

The spectinomycin resistance gene from pDMG4 [58] was amplified by PCR using the primers: 5′Spec-cass-NotI (ATAAGAATGCGGCCGCCGATTTTCGTTCGTGAATACATG) and 3′ Spec-cass-EcoRI (CGGAATTCCATATGCAAGGGTTTATTGTTT), digested with NotI-EcoRI and ligated into NotI-EcoRI digested pmCherry (Clontech) underlined sequences indicate NotI and EcoRI restriction sites. The 3105 bp region containing the pUC origin of replication, plac:mCherry and the spectinomycin resistance gene were PCR amplified using the primers: 5′pMcher-trunc (GAAGATCTGACCAAGTTTACTCATATATACT) and 3′ Spec-cass-EcoRI (CGGAATTCCATATGCAAGGGTTTATTGTTT). Underlined sequences indicate BglII and EcoRI restriction sites. This fragment was digested with BglII and EcoRI and ligated into the 2780 bp fragment from BglII-EcoRI digested pVT1461. The resulting plasmid (pMRKO-1, submitted to Genbank) is a suicide vector for *A. actinomycetemcomitans* and contains *oriT*, *mob*, and *tra* genes from pVT1461 along with the pUC origin of replication, mCherry expressed from plac, and a spectinomycin resistance cassette.

### Resting cell suspensions

*A. actinomycetemcomitans* was grown in CDM overnight either aerobically or anaerobically in the presence of 20 mM glucose or 40 mM L-lactate. Bacteria were then subcultured in 30 ml of medium and exponential phase cells (OD_600_ = 0.4) were collected by centrifugation (5,000 x g for 15 min) at 25°C. Cell pellets were resuspended in an equal volume of CDM lacking nucleotides, amino acids and any carbon source. Cells were incubated at 37°C aerobically or anaerobically depending on the test conditions for 1 hr. Cells were collected again by centrifugation as described above and resuspended to an OD_600_ of 2 in 3 ml of CDM without nucleotides, amino acids, pimelate and thioctic acid containing either 20 mM glucose or 40 mM lactate. Cells were incubated for 4 h at 37°C either aerobically or anaerobically. After incubation samples were stored at −20°C for HPLC analysis.

### D-Lactate assay

D-lactate assays were performed as described [30] with modifications. Glycylglycine buffer was replaced with an equal concentration of Bicine (Fisher, USA) buffer and enzymatic assays were monitored by spectrophotometry at 340 nm for 4 hours.

### Co-culture experiments

*A. actinomycetemcomitans* and *S. gordonii* were grown overnight in CDM containing 3 mM glucose. 3 mM glucose was used to ensure that the medium was limited for catabolizable carbon. Cells were diluted 1∶50 in the same medium and allowed to grow to exponential phase (OD_600_ of 0.2). Cells were then diluted 1∶100 (2×10^6^ *S. gordonii*/ml and 1×10^7^ *A. actinomycetemcomitans*/ml) as mono-cultures or co-cultures in 3 ml CDM containing 3 mM glucose. Cultures were allowed to grow for 10 h aerobically or 12 h anaerobically, after which cells were serially diluted, plated on either TSBYE agar + vancomycin for *A. actinomycetemcomitans* enumeration or TSBYE agar + streptomycin for *S. gordonii* enumeration. Colonies were counted after incubation at 37°C for 48 h. An aliquot of the culture was also stored at −20°C for HPLC metabolite analysis.

### HPLC analysis

Metabolite levels were quantified using a Varian HPLC with a Varian Metacarb 87H 300×6.5 mm column at 35°C. Samples were eluted using isocratic conditions with 0.025 N H_2_SO_4_ elution buffer and a flow rate of 0.5 ml/minute. A Varian refractive index (RI) detector at 35°C was used for metabolite enumeration by comparison with acetate, ethanol, formate, glucose, L-lactate, D-lactate, pyruvate and succinate standards.

### *In vivo* murine abscess growth

Murine abscesses were generated essentially as described previously [37]. Briefly, 6–8 week-old, female, Swiss Webster mice were anesthetized with an intraperitoneal injection of Nembutal (50 mg/kg). The hair on the left inner thigh of each mouse was shaved, and the skin was disinfected with 70% alcohol. Mice were injected subcutaneously in the inner thigh with 10^7^ CFU *A. actinomycetemcomitans*, *S. gordonii* or both. At 6 days post- infection, mice were euthanized and intact abscesses were harvested, weighed and placed into 2 ml of sterile PBS (or water for pH measurements). Tissues were homogenized, serially diluted and plated on Brain Heart Infusion (BHI) agar + 20 µg/ml Na_2_CO_3_ + vancomycin for *A. actinomycetemcomitans* enumeration or BHI agar + 20 µg/ml Na_2_CO_3_ + streptomycin for *S. gordonii* enumeration, to determine bacterial CFU/abscess. Experimental protocols involving mice were examined and approved by the Texas Tech University HSC Institutional Animal Care and Use Committee.

## Supporting Information

Text S1 Figure S1: Growth of *S. gordonii* in mono- or co-culture with *A. actinomycetemcomitans* or *A. actinomycetemcomitans lctD* ^-^ in aerobic and anaerobic co-cultures. Strains were grown as mono- or co-cultures in 3 mM glucose aerobically or anaerobically for 10 or 12 h respectively, serially diluted, and plated on selective media to determine colony forming units per ml (CFU/ml). *S. gordonii* mono-cultures numbers are represented by black bars, co-culture numbers with *A. actinomycetemcomitans* are represented by white bars, and co-culture numbers with *A. actinomycetemcomitans lctD* ^-^ are represented by grey bars. Error bars represent 1 standard error of the mean, n = 3. Figure S2: Survival of *S. gordonii* and *S. gordonii spxB* ^-^ in a murine abscess model. A. Number of bacteria recovered from each abscess expressed as colony forming units per abscess (CFU/abscess). Wilcoxon signed-rank test value, p<0.03. B. Abscess weights 6 days post-inoculation. Error bars represent 1 standard error of the mean, n = 4.
(DOC)
