# Supplementary figures and images for: Metabolite Cross-Feeding Enhances Virulence in a Model Polymicrobial Infection
Source: PLoS Pathog. 2011 Mar 31;7(3):e1002012. doi: 10.1371/journal.ppat.1002012 (PMC3069116; doi:10.1371/journal.ppat.1002012)

**Figure S1**

**
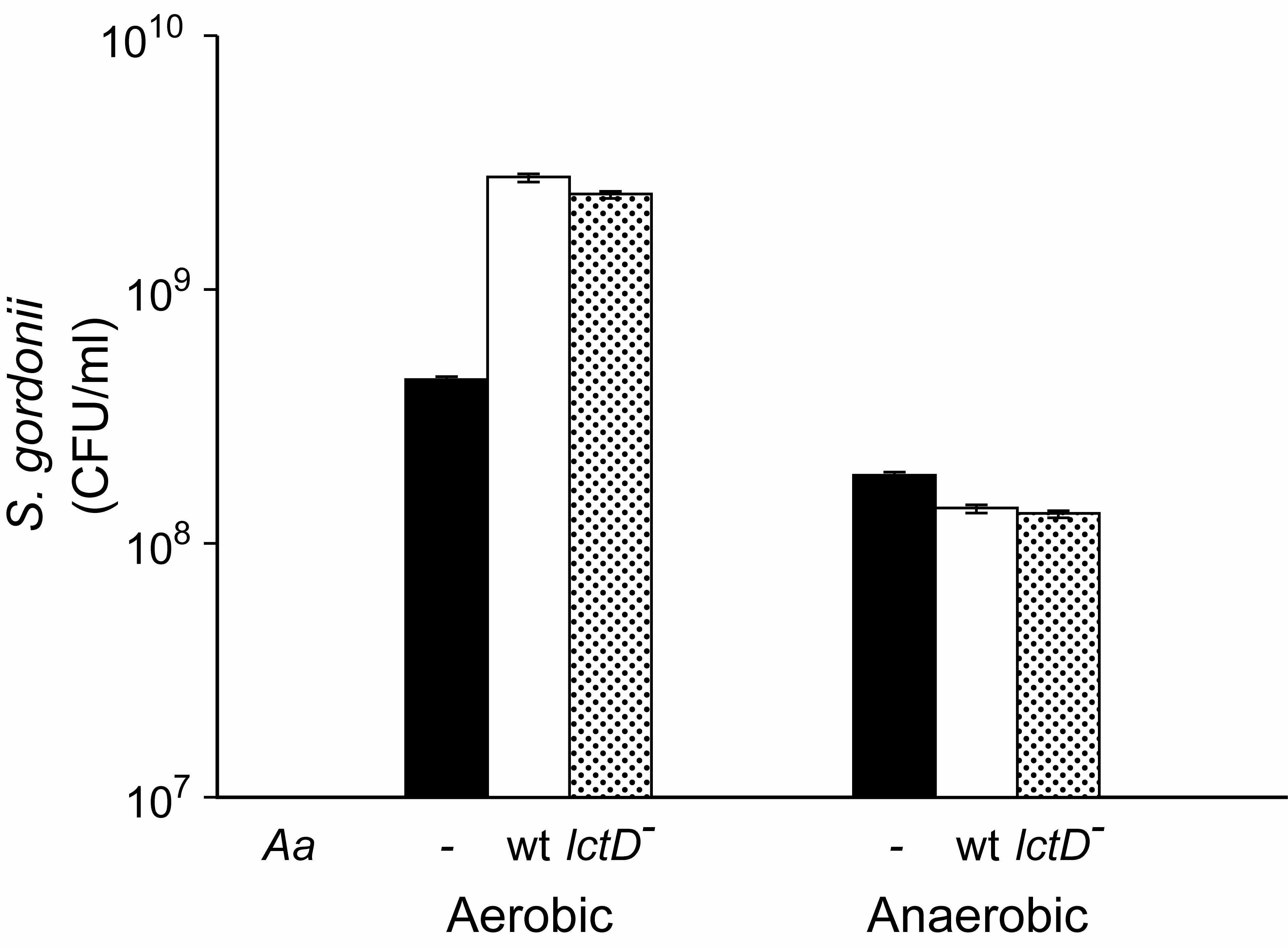
**

**Figure S2**


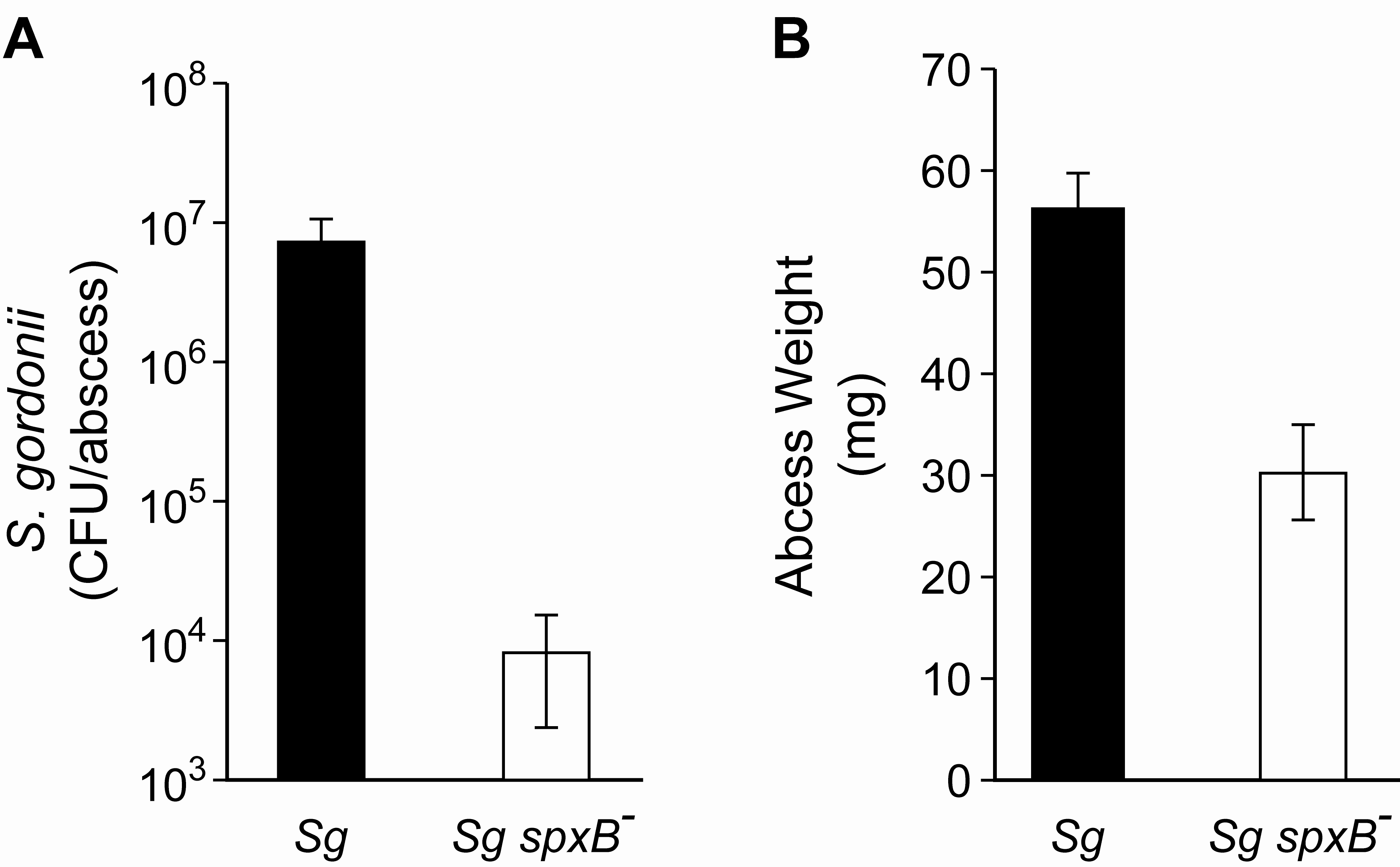

Supplement: Text S1 — Figure S1: Growth of S. gordonii in mono- or co-culture with A. actinomycetemcomitans or A. actinomycetemcomitans lctD - in aerobic and anaerobic co-cultures. Strains were grown as mono- or co-cultures in 3 mM glucose aerobically or anaerobically for 10 or 12 h respectively, serially diluted, and plated on selective media to determine colony forming units per ml (CFU/ml). S. gordonii mono-cultures numbers are represented by black bars, co-culture numbers with A. actinomycetemcomitans are represented by white bars, and co-culture numbers with A. actinomycetemcomitans lctD - are represented by grey bars. Error bars represent 1 standard error of the mean, n = 3. Figure S2: Survival of S. gordonii and S. gordonii spxB - in a murine abscess model. A. Number of bacteria recovered from each abscess expressed as colony forming units per abscess (CFU/abscess). Wilcoxon signed-rank test value, p<0.03. B. Abscess weights 6 days post-inoculation. Error bars represent 1 standard error of the mean, n = 4. (DOC) [file ppat.1002012.s001.doc]
